# A rapid test for protein–DNA interactions

**DOI:** 10.1093/nar/gkag142

**Published:** 2026-02-18

**Authors:** Casey J Toft, Holly M Radford, Alanna E Sorenson, Patrick M Schaeffer

**Affiliations:** Biomedical Sciences and Molecular Biology, College of Medicine and Dentistry, James Cook University, Douglas QLD 4811, Australia; Biomedical Sciences and Molecular Biology, College of Medicine and Dentistry, James Cook University, Douglas QLD 4811, Australia; Biomedical Sciences and Molecular Biology, College of Medicine and Dentistry, James Cook University, Douglas QLD 4811, Australia; Biomedical Sciences and Molecular Biology, College of Medicine and Dentistry, James Cook University, Douglas QLD 4811, Australia

## Abstract

The characterization of protein–DNA interactions underpinning fundamental biological processes requires methods that are laborious and time-consuming. Here, we introduce a rapid and instrument-free assay leveraging on GFP-tagged proteins and the lateral flow assay principle to examine protein–DNA interactions. The rapid protein–DNA interaction test (R-PNAI-T) detects complexes using a dipstick in ∼15 min. Validation of the R-PNAI-T with bacterial proteins involved in replication and transcription demonstrated its applicability for diverse protein–DNA interactions, achieving a remarkable detection sensitivity of ∼0.7 fmol with the *Escherichia coli* Tus protein. Analysis of these protein interactions with their specific target DNA sequences highlighted the capability of the R-PNAI-T to discern subtle differences in affinity, providing valuable comparative data. The R-PNAI-T is robust, retaining functionality in human serum and bacterial lysates. The assay showed tolerance towards biotin contaminants, expanding its use for quality control in protein purification processes and tracking complexes in biological samples. To demonstrate versatility, we applied the R-PNAI-T with polymerase chain reaction-amplified DNA to probe the putative origin of replication in *Burkholderia pseudomallei*, confirming a functional interaction between the DnaA initiator protein and the DnaA box in this bacterium. Overall, the R-PNAI-T is versatile, cost-effective, and user-friendly, offering broad applications in biological research and biotechnology.

## Graphical Abstract

**Graphical Abstract:**
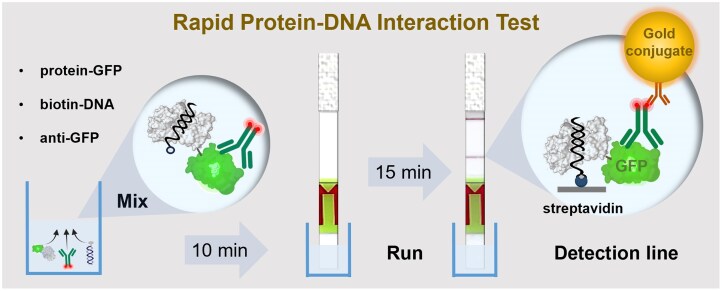

## Introduction

The interactions between proteins and nucleic acids are essential to biological processes such as DNA replication and repair, regulation of gene expression, gene silencing, splicing, transport, and translation. Uncovering the target preference and modality of binding of DNA- and RNA-binding proteins has been the focus of a plethora of studies to ascribe their function in cellular processes [1–4]. Over the past decades, numerous techniques have been developed to examine these interactions each with varying complexity and sensitivity [5–7].

In the 1970s, the nitrocellulose filter-binding assay was developed to identify protein-nucleic acid (PNA) binding [8]. This quasi-universal assay revolutionized the study of DNA–protein interactions by providing a sensitive and quantitative method to analyze the binding affinity between proteins and nucleic acids. The DNase I footprinting assay was first reported by David Galas and Albert Schmitz in 1978 [9]. This technique is used to identify DNA sequences to which a protein binds *in vitro*. It involves the digestion of DNA with DNase I enzyme, which cleaves DNA at sites where it is not protected by a bound protein. By comparing the cleavage pattern of DNA in the presence and absence of the protein, researchers can determine the specific DNA sequence protected by the protein. The DNAse protection assay has been widely used to study protein–DNA interactions and transcription factor binding sites [10, 11].

One of the earliest applications of the electrophoretic mobility shift assay (EMSA) was reported by Fried and Crothers in 1981 [12]. They used gel electrophoresis to study the binding of the lac repressor protein to DNA [12, 13]. The EMSA can be performed using either radioactively or fluorescently labelled DNA or through post-staining of DNA. It can provide information about the stoichiometry of the complex as well as DNA bending [14]. In crude samples where competing protein–DNA interactions can occur, derivatives of the technique such as the supershift assay [6, 15–17] and GFP-EMSA [18], are useful to visualize and confirm the formation of a specific complex between a protein and its target DNA. The EMSA is often used for qualitative purposes and scouting experiments due to its simplicity, low cost, and rapid turnaround in a few hours prior to interrogating a protein–DNA interaction using a more complex and informative technique. Similar techniques were developed for RNA-binding proteins and were of paramount importance to a multitude of studies over the following decades.

The first chromatin immunoprecipitation (ChIP) experiment was reported by Gilmour and Lis in 1984 [19]. They used a variation of immunoprecipitation techniques to study the association of RNA polymerase II with DNA in isolated nuclei from *Drosophila melanogaster* embryos. In their study, they employed an antibody against RNA polymerase II to immunoprecipitate the protein–DNA complexes from the isolated nuclei. This groundbreaking experiment laid the foundation for all ChIP techniques, which have since become a widely used method for studying protein–DNA interactions in eukaryotes and bacteria [20–27].

The next decade saw major developments fuelled by the availability of new technologies and instruments. The yeast one-hybrid (Y1H) principle was first reported in 1991 by Wilson *et al.* [28] for the identification of DNA-binding sites, followed in 1993 by Wang and Reed [29] and Li and Herskowitz [30] for the identification of DNA-binding proteins. It is a genetic method that can be used to identify either unknown DNA-binding proteins that bind to a specific DNA sequence [31, 32] or unknown DNA sequences that are bound by a specific protein [28, 31]. The Y1H is similar to the yeast two-hybrid assay that identifies protein–protein interactions allowing interrogation of a library of candidates [33]. The use of surface plasmon resonance (SPR) to study protein–DNA interactions started in the same period [34, 35]. One early example is a study published in 2000 by Neylon *et al.* [36]. In this work, the authors utilized SPR to investigate the binding of Tus to *Ter*. SPR yields equilibrium and kinetic data to dissect complex interactions, such as the unique transition of the Tus–Ter complex into a locked form [1, 37]. A multitude of fluorescence-based techniques have also been developed with the benefit of easier and faster protocols as well as greater throughput [38–43].

While most of these early and newer techniques have since been translated into high-throughput formats or even genome-wide approaches [5–7, 21, 44–46], they remain expensive, time-consuming, difficult to perform, and require complex instruments and analyses. Validating the DNA target is crucial for confirming ChIP-seq data, particularly when characterizing a protein *de novo*. Conducting feasibility studies are equally important before proceeding with single-molecule analyses [47, 48] and SPR experiments [37]. Notably, the initial stages of assay development, including optimization of buffer conditions, incubation times, and concentrations to estimate complex affinity and stability, are often the most challenging and time-consuming [49].

**Figure 1.**
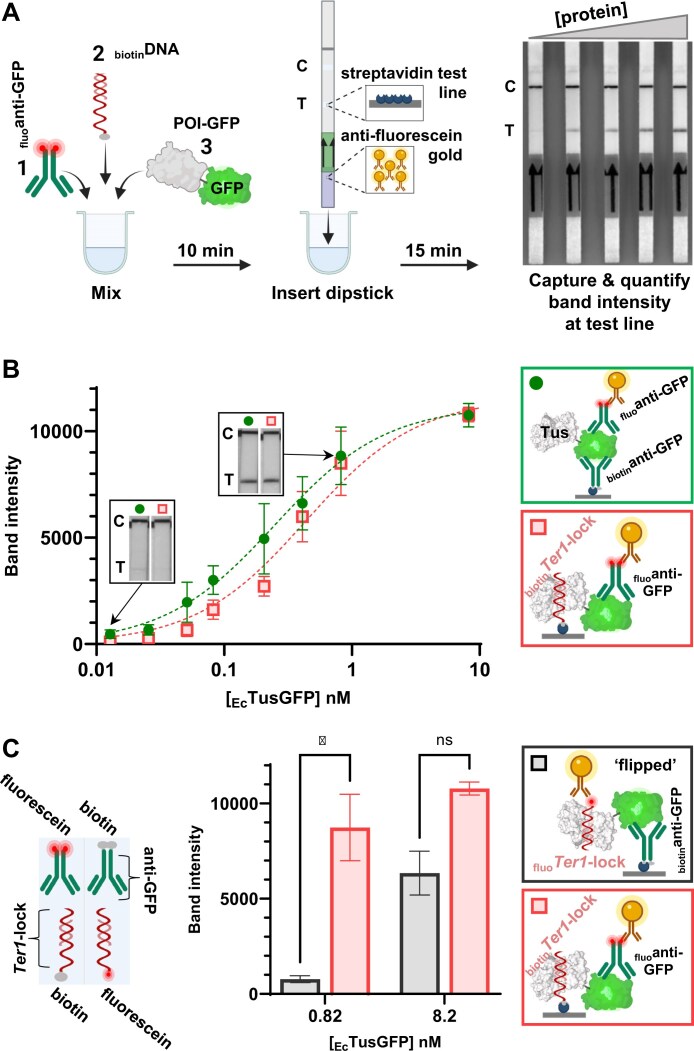
(**A**) General R-PNAI-T workflow using a Hybridetect dipstick consisting of anti-fluorescein conjugated gold nanoparticles and a streptavidin test line. A fluorescein-labelled antibody (_fluo_anti-GFP), biotinylated DNA (_biotin_DNA), and a GFP-tagged protein (POI-GFP) are simply mixed (see Supplementary Information step-by-step procedure). After 10 min, a Hybridetect dipstick is inserted for 15 min. (**B**) Comparison of _Ec_TusGFP detection with an anti-GFP antibody pair (_biotin_anti-GFP/_fluo_anti-GFP, green circles) or _biotin_*Ter1*-lock and _fluo_anti-GFP (red squares). Insert images correspond to detectable test lines for 0.7 fmol (0.013 nM) and 45 fmol (0.82 nM) of _Ec_TusGFP. (**C**) Comparison of *Ter1*-lock capture format (_biotin_*Ter1*-lock and _fluo_anti-GFP, red columns) and ‘flipped’ *Ter1*-lock detection format (_biotin_anti-GFP and _fluo_*Ter1*-lock, black columns) for _Ec_TusGFP (*P*-values are < 0.05). A schematic key comparing key component modifications for the different formats is provided (bottom left). Band intensities were quantified using ImageJ. Partially created in BioRender. Toft, C. (2026) https://BioRender.com/w35o738.

Our goal was to develop the first instrument-free, rapid protein–DNA interaction test (R-PNAI-T) with quasi-universal applications in specialized laboratories, educational settings, and low-resource areas. Our assay is built upon the lateral flow assay principle [50] that has dominated the diagnostic market in recent years with picomolar analyte detection capability as seen with the SARS-CoV-2 nucleocapsid protein (NP) [51–53]. The R-PNAI-T principle involves the capture and immunodetection of a GFP-tagged DNA-binding protein of interest (POI) in complex with a biotinylated target oligonucleotide using a commercially-available Hybridetect dipstick (Fig. 1A). The test can be run in <30 min. Several GFP-tagged DNA-binding proteins were chosen to validate and explore the capability of our R-PNAI-T. Two bacterial replication terminator proteins (Tus) from *Dickeya paradisiaca* and *Escherichia coli* [54], and a bifunctional group II biotin protein ligase (BirA), which also acts as a transcriptional repressor of the biosynthesis pathway in *E. coli* [55], were used for validation of the rapid test and to explore its limits. The rapid test was then adapted to investigate the binding of the putative initiator protein DnaA to specific target sequences within the predicted origin of replication in chromosome I of *Burkholderia pseudomallei*. Overall, the R-PNAI-T is robust and reliable, yielding reproducible and comparative data for our selection of proteins with diverse DNA-binding properties.

## Materials and methods

### Resources and proteins

See Supplementary Information for list of antibodies and oligonucleotide sequences. GFP-tagged proteins used in this study include: *Dickeya paradisiaca *_Dp_TusGFP [54], *E. coli *_Ec_TusGFP [54], *E. coli* BirA-GFP [56], SARS-CoV-2 NP-GFP [52], MERS-CoV NP-GFP [52], *B. pseudomallei *_Bp_DnaG-GFP [40, 57], and _Bp_DnaA-GFP. Cloning, protein expression, and purification followed established procedures (details in Supplementary Information) [41]. Briefly, all proteins were expressed with an N-terminal hexahistidine tag and a C-terminal GFP tag and purified by nickel affinity column chromatography.

### R-PNAI-T: Tus–*Ter* and Tus–*Ter*-lock binding

Binding of Tus-GFP proteins to *Ter* DNA species (see Supplementary Table S1 for oligonucleotide sequences) was examined using Hybridetect dipsticks (Milenia Biotec). Oligonucleotides were typically resuspended in phosphate buffered saline (PBS) (pH 7.4) and annealed at 10 µM final concentration using a short 2–5 min incubation at 95°C in a heating block, followed by slow cooling to room temperature unless otherwise stated (see Supplementary Table S1). In a 96 well-plate, 5 µl of fluorescein-labelled anti-GFP antibody (see Supplementary Table S1, _fluo_anti-GFP) diluted 1:500 in PBS + 20% (w/v) sucrose were mixed with 5 µl of 1 µM _biotin_*Ter1* or _biotin_*Ter1*-lock diluted in PBS. Then, 45 µl _Dp_TusGFP or _Ec_TusGFP at concentrations ranging from 100 nM to 15.6 pM (obtained with a 10-fold dilution series from 100 to 1 nM and a 2-fold dilution series from 500 to 15.6 pM diluted in PBS) were added. Control reactions without TusGFP or without _biotin_*Ter*, were also prepared. After 10 min at room temperature, Hybridetect dipsticks were placed into wells for 15 min. Results were captured with a gel documentation system (G:BOX Chemi XRQ) using 150 ms exposure on a laminated A4 sheet containing a ‘printed line’ (HEX #A0A0A0). Band intensities were quantified using ImageJ with the ‘printed line’ used for normalization as previously described [51]. The results were compared to anti-GFP sandwich controls in which _biotin_*Ter* was replaced with a biotin-labelled anti-GFP antibody (see Supplementary Table S1, _biotin_anti-GFP) diluted 1:500 in PBS + 20% (w/v) sucrose. A detailed step-by-step procedure from sample preparation to data analysis is available in the Supplementary Information.

### Competitive R-PNAI-T

In a 96 well-plate, 5 µl _biotin_*Ter1*-lock (5 nM) was mixed with 0.5 µl untagged *Ter1 or Ter1*-lock (5 µM, i.e. 100-fold excess over _biotin_*Ter1*-lock) and 5 µl of 1:500 _fluo_anti-GFP. Then, 45 µl of 10 nM _Dp_TusGFP or _Ec_TusGFP was added. After 10 min, Hybridetect dipsticks were inserted, and results were captured and quantified as above. Control reactions without competitor were also run.

### R-PNAI-T: bacterial cell lysate and human serum interference

BL21(DE3)RIPL bacteria were cultured on LB agar with chloramphenicol at 37°C overnight. A single colony was used to inoculate 5 ml of LB chloramphenicol broth, which was then shaken at 200 RPM and 37°C overnight. A 100 μl aliquot of the culture was transferred into 5 ml of fresh LB chloramphenicol broth and incubated until the culture reached an OD600 of 0.8. The culture was then shifted to 16°C for an additional 48 h. Cells were harvested by centrifugation at 4°C and 3000 × *g* for 20 min. The cell pellet was resuspended in ice-cold lysis buffer [50 mM sodium phosphate (pH 7.8), 300 mM NaCl, 10% (v/v) glycerol, 2 mM β-mercaptoethanol and 1 mg/ml lysozyme] at 7.5 ml/g of wet cell pellet and disrupted by sonication on ice using a probe sonicator (20 kHz, three 10 s pulses with 30 s cooling intervals). The lysate was then centrifuged at 4°C and 40 000 × *g* for 20 min.

Briefly, 5 µl of 10 nM _Ec_TusGFP (50 fmol, diluted in PBS) were mixed with 45 µl of diluted bacteria lysate supernatant (i.e. 22.5 µl lysate mixed with 22.5 µl of PBS) or 45 µl neat human AB serum, followed by 5 µl of 1:500 _fluo_anti-GFP [diluted in PBS + 20% (w/v) sucrose] and 5 µl of 1 µM _biotin_*Ter1*-lock (5 pmol diluted in PBS). After 10 min, Hybridetect dipsticks were inserted and results were captured and quantified as above. Lysate and serum control reactions without TusGFP were performed.

### R-PNAI-T: biotin tolerance and BirA-*bioO* binding

The effect of free biotin was first evaluated with a sandwich format, including 10 µl of 20 nM BirA-GFP (diluted in PBS or PBS with 1 µM biotin), 20 µl of PBS containing 1:1000 _fluo_anti-GFP and 1:1000 _biotin_anti-GFP, as well as 10 µl of annealing buffer (10 mM Tris, pH 8.0, 150 mM NaCl). Binding of BirA-GFP to *bioO* (see Supplementary Table S1 in Supplementary Information) was examined in various conditions. Briefly, 10 µl of 1 µM _biotin_*bioO* (annealing buffer) was mixed with 10 µl of 20 nM BirA-GFP in: PBS; PBS with 1 µM biotin; or PBS with 1 µM biotin, 1 mM ATP and 1 mM MgCl_2_. Then, 20 µl of 1:1000 _fluo_anti-GFP was added to the reactions. After 10 min, Hybridetect dipsticks were inserted in reaction wells and results were captured and quantified as above. Control reactions without BirA-GFP or _biotin_*bioO*, as well as control reactions with GFP instead of BirA-GFP were also performed.

### R-PNAI-T: identification of DnaA boxes in PCR amplicons

Polymerase chain reaction (PCR) primers were designed to produce amplicons that are similar in size (PCRa and PCRb; see Supplementary Information for *OriC* candidate identification). PCR (25 µl) were run using Immomix (Bioline) containing 1 µl genomic DNA (*B. pseudomallei* K96243) with 500 nM forward and reverse primers. PCRa and PCRb concentrations were adjusted with PBS (pH 7.4) after quantification by agarose gel electrophoresis and ImageJ integration. Briefly, 5 µl PCR, 5 µl of 1:500 _fluo_anti-GFP, and 45 µl of 100 nM _Bp_DnaA-GFP (PBS, 5% glycerol, 5 mM MgCl_2_, pH 7.2) were incubated 5 min at RT. Hybridetect dipsticks were inserted and results were captured and quantified as above. ‘No template’ controls were performed.

### Statistical analysis

Analyses were performed using GraphPad Prism 10. Repeat number ≥ 2. Unpaired *t*-test was used to compare the difference in band intensity obtained with _biotin_*Ter1*-lock capture and _biotin_anti-GFP capture. Binding curves were fitted with the Hill equation to identify possible deviation from a standard hyperbolic binding curve, then again with Hill coefficient set to 1.

## Results and discussion

### Development and validation of the R-PNAI-T

Lateral flow assay technology has been around for more than six decades [50], allowing sensitive detection of protein analytes. The unprecedented development of rapid tests during the SARS-CoV-2 pandemic pushed their analytical capabilities to the limit [58]. Indeed, some brands of COVID-19 rapid tests can reliably detect the presence of NP at low picomolar concentrations [51–53]. At the other end of the lateral flow assay spectrum, we find the nucleic acid lateral flow immunoassay (NALFIA) designed to detect biotin- or fluorescein(fluo)-labelled LAMP/PCR amplicons [59–61]. Leveraging the NALFIA platform, we designed a groundbreaking, rapid test for the detection of protein–DNA interactions. The simple experimental setup of our R-PNAI-T is shown in Fig. 1A, where a biotin-labelled DNA is mixed with a GFP-tagged POI and a fluorescein-labelled antibody specific for GFP (_fluo_anti-GFP). Next, a Hybridetect dipstick is added to a well containing the mixture for 15 min, and a detectable band appears at the test line, indicating the formation of a protein–DNA complex. A detectable band forms when the ternary complex is captured by streptavidin immobilized at the test line and bound by gold nanoparticles conjugated with anti-fluorescein antibodies, which are integral to the dipstick.

Initially, we evaluated our R-PNAI-T with Tus, which is one of the strongest monomeric DNA-binding proteins [62]. Tus plays a crucial role in DNA replication by binding to *Ter* sites, and even more strongly to the *Ter*-lock structure generated by the action of the replicative helicase [1, 21, 37, 45, 46, 54, 63]. We compared the band intensity obtained from the binding of _Ec_TusGFP to _biotin_*Ter1*-lock DNA derived from *D. paradisiaca* [54] using _fluo_anti-GFP for detection (Fig. 1B, red squares), to the band intensity obtained from the binding of a _biotin_anti-GFP and _fluo_anti-GFP pair to the GFP tag (Fig. 1B, green circles). The _biotin_anti-GFP/_fluo_anti-GFP pair was used to establish a reference limit of detection based on antibody affinity and optical density of the gold nanoparticles for the test strip. In the moderate salt and pH conditions of PBS, _biotin_*Ter1*-lock and _biotin_anti-GFP yielded similar concentration-dependent band intensities with _Ec_TusGFP (Fig. 1B). No detectable test lines could be observed when _Ec_TusGFP or _biotin_*Ter1*-lock were omitted confirming that the R-PNAI-T is not prone to non-specific binding (Supplementary Fig. S1, top panel). Of note, the anti-GFP sandwich format was applied to various GFP-tagged proteins (Supplementary Fig. S1, bottom panel). As expected, the test strips produced highly similar concentration-dependent band intensities (Supplementary Fig. S1) highlighting the robustness and reproducibility of the test strips. When coupled with densitometry, the R-PNAI-T could detect ∼0.7 fmol _Ec_TusGFP in a sample with the _biotin_*Ter1*-lock capture format (Fig. 1B).

Next, we examined the performance of the R-PNAI-T in a ‘flipped’ format. Here, the detection of the _Ec_TusGFP-*Ter1*-lock complex was evaluated using _biotin_anti-GFP/ _fluo_*Ter1*-lock (Fig. 1C, grey). A significant loss in detection sensitivity was evident compared to _biotin_*Ter1*-lock/_fluo_anti-GFP (Fig. 1C). In fact, the band intensity obtained with 45 fmol _Ec_TusGFP using _fluo_*Ter1*-lock detection (cf. ∼770 AU at 0.82 nM in Fig. 1C) is comparable to the one obtained with 2.8 fmol _Ec_TusGFP using _biotin_*Ter1*-lock capture (cf. ∼665 AU at ∼51 pM in Fig. 1B). The _fluo_*Ter1*-lock is a 6-FAM derivative, compared with a 6-FITC derivative for the _fluo_anti-GFP. As these fluorescein derivatives only differ in their coupling groups, it is unlikely this would significantly affect the detection sensitivity of the anti-fluorescein gold conjugate (Supplementary Fig. S2). Instead, we suspect this loss in detection sensitivity is primarily due to a difference in the number of *Ter1*-lock and anti-GFP molecules as well as the number of fluorescein on these, i.e. one FAM molecule per _fluo_*Ter1*-lock compared to multiple FITC on the _fluo_anti-GFP. Thus, the ‘flipped’ R-PNAI-T format was not investigated further.

The R-PNAI-T validation data highlighted a robust test line repeatability and a broad 3-log dynamic range, which are crucial attributes for quantifying protein–DNA interactions. Test lines can simply be photographed or scanned for quantitative analysis and record keeping. Advantageously, once dried, the test lines are very stable. The analytical sensitivity was significantly better with the biotinylated DNA capture format of the R-PNAI-T than with the ‘flipped’ format and was not affected by any background signal at the test line. The biotinylated DNA capture format made it possible to detect protein–DNA complex formation using just 0.7 fmol of protein (see Fig. 1B, *Ter1*-lock capture). This sensitivity is comparable to instrument-based assays such as EMSA, supershift assay, and SPR. Results are obtained in <30 min making the R-PNAI-T the fastest and easiest protein–DNA binding assay to perform.

### Comparison of Tus–*Ter* and Tus–*Ter*-lock interactions

**Figure 2.**
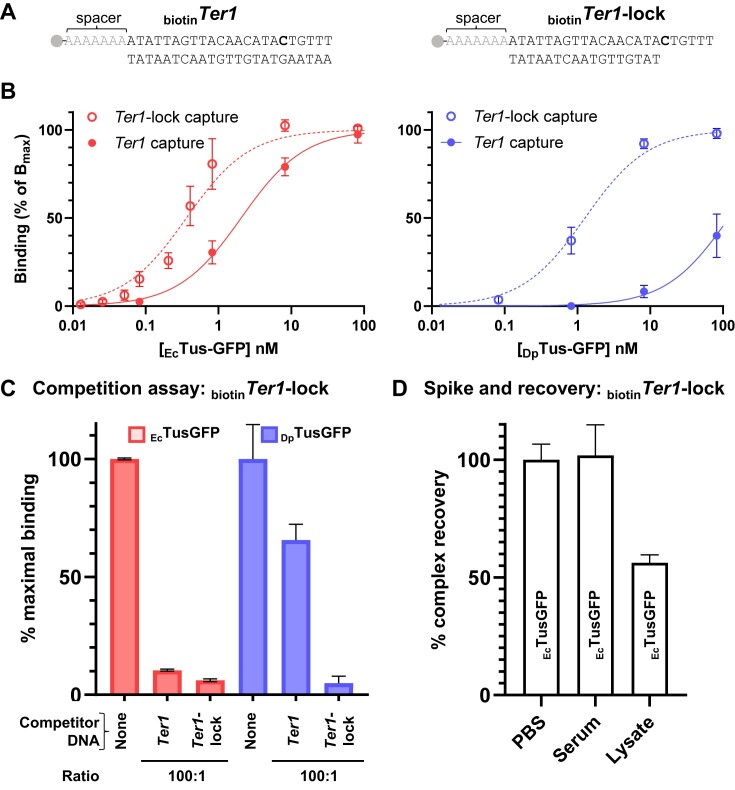
Comparison of *E. coli* and *D. paradisiaca* Tus–Ter and Tus–Ter-lock detection. (**A**) Details of _biotin_*Ter1* and _biotin_*Ter1*-lock sequences. The 5′-biotin is followed by a poly(A) spacer sequence that separates *Ter1* from the streptavidin surface, minimizing potential steric interference between Tus and the surface. The unpaired C (6) (bold) locks into the known cytosine-binding pocket observed in the Tus–Ter-lock crystal structure [1]. (**B**) Comparison of the R-PNAI-T binding profiles of _Ec_TusGFP (red) or _Dp_TusGFP (blue) with _biotin_*Ter1* or _biotin_*Ter1*-lock. Protein concentrations ranged from 82 nM to 12.8 pM. (**C**) Competitive R-PNAI-T for _Ec_TusGFP (red) and _Dp_TusGFP (blue) with _biotin_*Ter1*-lock in the presence of 100-fold excess *Ter1* or *Ter1*-lock competitor. (**D**) Spike-recovery experiments with 50 fmol _Ec_TusGFP and 5 pmol _biotin_*Ter1*-lock in neat human serum or bacterial cell lysate (15 ml/g wet bacteria) compared to PBS. Band intensities were quantified using ImageJ. The percentage complex recovery is calculated by dividing the test line band intensity obtained in PBS (control experiment) with the band intensity obtained in serum or lysate, and expressed as a percentage value.

The binding of _Ec_TusGFP and _Dp_TusGFP to the canonical *Ter* and *Ter*-lock sequences has been thoroughly examined with a variety of techniques, such as SPR, differential scanning fluorimetry of GFP-tagged Proteins (DSF-GTP), and ChIP-seq, yielding a large body of comparative affinity data [1, 21, 37, 45, 46, 63]. While the binding kinetics of _Ec_TusGFP with *Ter*-lock and *Ter* are significantly different [1], their equilibrium constants are quite similar [37]. In stark contrast, the binding of _Dp_TusGFP to *Ter1*-lock is ∼50-fold stronger than to *Ter1* [54]. This intriguing difference in binding behaviour is due to the absence of 6 nucleotides in the *Ter1*-lock bottom strand sequence, which is mimicking the unzipping action of the replicative helicase by exposing a cytosine residue that locks into a binding pocket of Tus (Fig. 2A) (1). As such, comparing the binding of these protein–DNA complexes presented an ideal model to further evaluate the capability of the R-PNAI-T to capture differences in affinity.

We compared the binding profiles of _Ec_TusGFP and _Dp_TusGFP with _biotin_*Ter1* or _biotin_*Ter1*-lock sequences under identical temperature and buffer conditions with varying TusGFP and fixed _biotin_*Ter* DNA concentrations (Fig. 2B). Here, the 50% binding value (B_50_) is used to determine the concentration (BC_50_) of analyte (protein or DNA) yielding 50% of the maximum band intensity for a protein–DNA complex. BC_50_ is an apparent affinity measure that can be used to rank binding strengths across samples but should not be directly interpreted as *K*_D_, especially with very tight interactions [64]. In our conditions, we can rank the affinity of TusGFP for *Ter* species and report the minimum fold-difference in *K*_D_ between complexes. Maximum band intensity (B_max_) values of ∼10 500 were observed for _Ec_Tus-GFP with both _biotin_*Ter1* and _biotin_*Ter1*-lock, and for _Dp_TusGFP with _biotin_*Ter1*-lock, whereas no comparable saturation was reached with _biotin_*Ter1*. A BC_50_ value of 0.36 nM was determined for the most stable _Ec_TusGFP–*Ter1*-lock complex, which is ∼5-fold more stable than the _Ec_TusGFP–*Ter1* complex (BC_50_ = 2.05 nM) in moderate salt conditions (Fig. 2B). In contrast, for _Dp_TusGFP, the BC_50_ values differed by ∼100-fold between *Ter1*-lock (BC_50_ = 1.27 nM) and *Ter1* (BC_50_ = 121 nM). The BC_50_ obtained with _Dp_TusGFP and *Ter1* highlighted the limits of the R-PNAI-T for quantitative applications. Here, the top plateau value of the hyperbolic fit was fixed at 100% as saturation was not reached with 82 nM _Dp_TusGFP. The *R*^2^ value of 0.88 obtained for this fit stood in stark contrast to the three other datasets, which showed excellent *R*^2^ values ranging from 0.946 to 0.987. Nevertheless, the 100-fold difference in BC_50_ between *Ter1* and *Ter1*-lock is consistent with previous _Dp_TusGFP data [54]. Of note, increasing the protein–DNA complex formation time from 10 to 120 min did not significantly affect the R-PNAI-T results, ensuring that the difference in _Dp_TusGFP binding to *Ter1*-lock and *Ter1* was not due to an insufficient equilibration time (Supplementary Fig. S3). Our results indicate that the R-PNAI-T B_max_ is governed by detection limits associated with the protein, antibody, and gold nanoparticle rather than by DNA availability. As TusGFP concentration increases, each gold nanoparticle-anti-GFP conjugate can engage multiple protein–DNA complexes, but only a subset of these interactions are efficiently captured on the streptavidin surface. Moreover, the finite number of accessible anti-GFP binding sites and nanoparticles constrains the total detectable signal. Consequently, the assay operates under protein- and detection-limited conditions, and the measured BC_50_ reflects the protein concentration required to reach half-maximal detectable signal rather than true equilibrium binding. This allows reliable ranking of relative affinities but prevents direct determination of absolute *K*_D_ values.

To validate the R-PNAI-T data reflecting differences in binding affinities, a competitive binding assay was performed using a 100-fold excess of non-biotinylated *Ter* sequences (*Ter1* or *Ter1*-lock) over the _biotin_*Ter1*-lock in the presence of either _Ec_TusGFP or _Dp_TusGFP (Fig. 2C). As expected for _Ec_TusGFP and _Dp_TusGFP, the *Ter1*-lock competitor almost completely outcompeted the _biotin_*Ter1*-lock yielding ∼95% reduction in test band intensity. The *Ter1* competitor yielded a 90% reduction in band intensity with _Ec_TusGFP. In contrast, _Dp_TusGFP retained ∼65% of its band intensity with the *Ter1* competitor (Fig. 2C) confirming our direct R-PNAI-T data (Fig. 2B). These competition assays corroborate the direct R-PNAI-T results, demonstrating that both _Ec_TusGFP and _Dp_TusGFP specifically bind *Ter1*-lock with high affinity.

The nearly complete displacement of _biotin_*Ter1*-lock by the *Ter1*-lock competitor (∼95% displacement for both proteins) confirms the specificity and robustness of the assay. In contrast, the reduced competition observed with the *Ter1* sequence reveals a clear difference between the two proteins. While _Ec_TusGFP binds *Ter1* and *Ter1*-lock with almost comparable affinity, _Dp_TusGFP shows a pronounced preference for *Ter1*-lock. These findings indicate that _Dp_TusGFP discriminates more strongly between *Ter1* and *Ter1*-lock, consistent with a 100-fold higher selectivity for the *Ter1*-lock. The R-PNAI-T can be employed in a direct or competitive format to produce relevant affinity data allowing easy comparison of different protein–DNA interactions. The experimental conditions are relatively flexible and the concentrations of protein, as well as biotinylated DNA and competitor can be varied by several orders of magnitude (Supplementary Fig. S4 and Fig. 2).

### Robustness of the R-PNAI-T

Matrix effects and contaminants can significantly affect the accuracy, sensitivity, and reliability of separation techniques. Here, we examined the tolerance of the R-PNAI-T towards crude samples such as bacteria lysates and human serum to determine if it could be useful to follow the production of a DNA-binding protein or its fate in biotechnological applications such as in non-viral gene delivery studies. Spike and recovery experiments were conducted with _Ec_TusGFP and _biotin_*Ter1*-lock spiked into neat human serum or clarified *E. coli* lysate, and compared with PBS (Fig. 2D). The test line band intensity of the R-PNAI-T was not affected in neat human serum (Fig. 2D), albeit there were some distinguishable differences in capillary flow rate due to the high viscosity of the sample. Of note, the absence of serum interference has previously been shown with this particular GFP-antibody pair by immuno-PCR [65]. In contrast, only 60% recovery was attained with the *E. coli* lysate. This loss in detection sensitivity is likely the result of a highly competitive binding environment, i.e. the high abundance of DNA binding proteins and target sites (i.e. endogenous Tus and *Ter* sites), as well as the higher ionic strength and pH of the lysis buffer compared with PBS. Importantly, both the human serum and *E. coli* lysate yielded a negative test line when _Ec_TusGFP was omitted (Supplementary Fig. S5).

**Figure 3.**
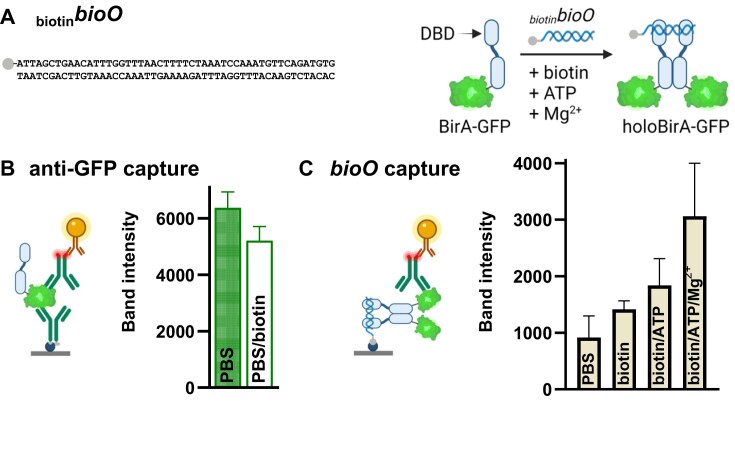
R-PNAI-T performance with BirA-GFP in the presence of biotin. (**A**) Biotinylated *bioO* target sequence (_biotin_*bioO*). BirA-GFP forms a high-affinity holoenzyme complex (holoBirA-GFP) with _biotin_*bioO* in the presence of biotin, ATP, and Mg^2+^. (**B**) Effect of biotin (250 nM) on the detection of BirA-GFP (5 nM) with an anti-GFP antibody pair (_biotin_anti-GFP/_fluo_anti-GFP). (**C**) Binding of BirA-GFP (5 nM) to _biotin_*bioO* in PBS or various combinations of biotin, ATP, and Mg^2+^. Band intensities were quantified using ImageJ (see Supplementary Information step-by-step procedure). Partially created in BioRender. Toft, C. (2026) https://BioRender.com/m08n796.

A multitude of cell culture conditions and purification methods may contain or use biotin. As the R-PNAI-T is dependent on a biotinylated oligonucleotide, we evaluated its tolerance towards this ubiquitous reagent and possible contaminant to determine its suitability for use in settings where biotin may be present. For this, we selected BirA, which forms a high-affinity complex with *bioO* in the presence of biotin, ATP, and magnesium ions (Fig. 3A). These substrates and cofactor are essential for formation of the dimeric BirA-biotinyl-5′-AMP holoenzyme complex [56] and its gene regulatory function [55]. First, we established the reference test line band intensity for 5 nM BirA-GFP with varying concentrations of _biotin_anti-GFP/_fluo_anti-GFP antibody pair in the absence of biotin (Supplementary Fig. S6). Then, we repeated this test in non-plateau conditions (anti-GFP pair dilution of 1:2000) in the presence of 250 nM biotin, which yielded ∼20% reduction in band intensity (Fig. 3B). This reduction in band intensity was deemed acceptable to compare the effect of biotin alone or biotin with ATP and Mg^2+^ on the binding of BirA-GFP to _biotin_*bioO* (Fig. 3C). The R-PNAI-T data clearly show enhanced binding of BirA-GFP to _biotin_*bioO* in the presence of biotin, ATP, and Mg^2+^. This is consistent with previous studies demonstrating that the BirA holoenzyme dimer exhibits the highest affinity for *bioO*, as well as a recent report showing that the stability of the *bioO*-BirA-GFP is reduced when only ATP and biotin are present compared with the full combination of ATP, biotin and Mg^2+^ [49, 56, 66].

The robust detection capability of the R-PNAI-T in human serum, as well as in competitive binding environments highlight a potential use in non-viral gene delivery studies, e.g. biodistribution and blood clearance of protein–DNA complexes [67–69]. Further quality control applications are envisaged during protein purification processes. Note here that the tolerance of the R-PNAI-T for biotin contaminants will depend on the concentration of biotinylated oligonucleotide used for capture.

### Putative origin of replication in *Burkholderia pseudomallei*

**Figure 4.**
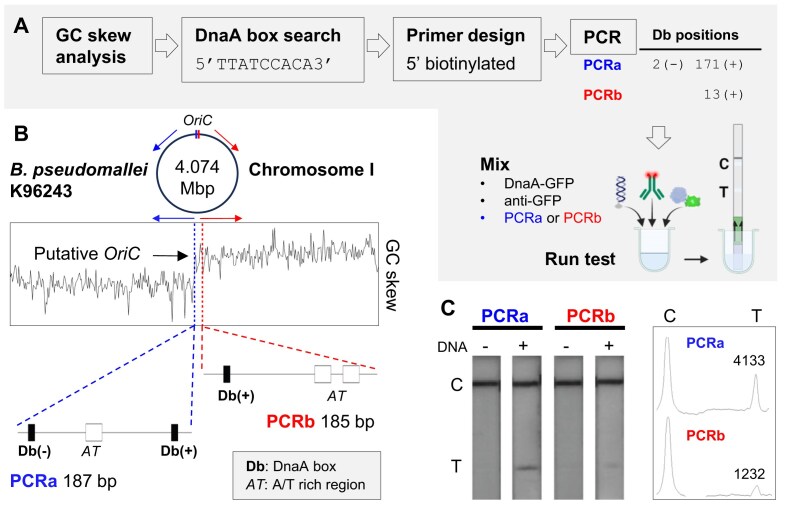
(**A**) Workflow for the identification and characterization of DnaA boxes (Db) in PCR amplicons (PCRa and PCRb) obtained from *B. pseudomallei*. Db positions are indicated relative to PCR products: (+) indicates the consensus sequence is on the top strand, and (−) on the bottom strand in the genomic context. Numbers indicate the start (+) or end (−) of the consensus sequence relative to the top strand (see Supplementary Table S1 for primer sequences and genomic coordinates). (**B**) GC skew analysis and BLAST search on chromosome 1 (GenBank ID: BX571965.1). Two regions were selected containing one or two DnaA boxes for PCR amplification (PCRa and PCRb). (**C**) Test lines for PCRa and PCRb as well as ‘no template’ controls (−) with _Bp_DnaA-GFP and _fluo_anti-GFP. Test lines were integrated using ImageJ. Partially created in BioRender. Toft, C. (2026) https://BioRender.com/w35o738.

To demonstrate the versatility of our assay, we applied the R-PNAI-T to probe the putative origin of replication in *B. pseudomallei* with the predicted _Bp_DnaA initiator protein. We identified several sequences corresponding to the canonical DnaA box (Fig. 4A) found in other bacteria [23, 70–73] around the two chromosomes of *B. pseudomallei*. We identified 13 fully matching sequences on chromosome I and 2 on chromosome II, along with numerous additional degenerate sequences. We focused on two small regions containing these sequences located at or near the GC skew switch, which typically marks the transition between leading and lagging strand synthesis, and thus indicates the location of the origin of replication [74–76]. These regions were amplified by PCR with one of the primers bearing a 5′-biotin for capture at the dipstick streptavidin test line. One amplicon contained two identical DnaA box sequences in opposite orientation (Fig. 4B, PCRa), as well as one degenerate sequence (two mismatches). The other amplicon (Fig. 4B, PCRb) contained only one full matched and one degenerate sequence (two mismatches). The PCRa amplicon clearly yielded the strongest test line band intensity with _Bp_DnaA-GFP (Fig. 4C). It is important to note that the _Bp_DnaA has not been studied previously. As such, our R-PNAI-T data provide the first experimental evidence of a functional interaction between DnaA and the DnaA box sequence in *B. pseudomallei*. Further investigation will be needed to fully map the binding and oligomerization sites in the *B. pseudomallei* orisome.

## Conclusions

The development of our instrument-free R-PNAI-T is a significant advancement in the field of protein–DNA interaction studies. The high sensitivity and robustness of the R-PNAI-T underscore its potential as a versatile tool for biological research, offering broad applications in molecular biology and biotechnology. It is very forgiving with respect to sample complexity and the presence of competitive binding environments. However, the R-PNAI-T has some limitations. While it excels in rapid semi-quantitative analysis and comparative studies of protein–DNA interactions, it does not provide the detailed kinetic insights offered by SPR [36, 37]. Additionally, despite the impressive picomolar sensitivity of the R-PNAI-T, it cannot compete with the analytical performance of a polyplex qPCR DNA-binding assay [45]. However, unlike the latter, the R-PNAI-T is not limited to double stranded DNA and yielded unique comparative *Ter*-lock data.

A major strength of the R-PNAI-T lies in its versatility and ease of use. Unlike traditional assays that require specialized equipment and lengthy protocols, our instrument-free R-PNAI-T streamlines the process. It delivers results in <30 min in a direct or competitive format, and without the need for extensive sample preparation. This accessibility makes it an attractive option for laboratories with limited resources or expertise, democratizing the study of protein–DNA interactions and expanding its reach to a wider user base. As such the R-PNAI-T has the potential to become a staple laboratory practical in molecular biology and biochemistry curricula at universities and even secondary schools.

The R-PNAI-T holds promise for the rapid screening of nucleic acid target sequences and their binding proteins in fields such as gene regulation, DNA repair, and RNA processing [5]. It enables comparative analysis of protein interactions with different nucleic acid sequences, shedding light on the specificity and dynamics of these molecular interactions. The tolerance of the R-PNAI-T towards matrix effects and biotin contaminants increases its utility in complex biological samples, opening avenues for applications in drug development, biotechnology, and personalized medicine. While its current format involves the production of a GFP-tagged protein, other common affinity tags and their specific antibodies, or even a protein-specific antibody, could be used instead. As such, the R-PNAI-T could be adapted for the detection of viral DNA- or RNA-binding proteins, such as the SARS-CoV NP [77], in clinical samples, offering a rapid and cost-effective alternative to traditional immunoassays. It could also be employed to assess and help the development of nucleic acid-based therapeutics involving high-affinity aptamer-protein interactions [78], or to validate inhibitors of key transcription factors implicated in cancer pathways [79].

While additional optimization and validation could further define its limits and scope, the R-PNAI-T already holds great potential to drive discovery, innovation, and ultimately advance our understanding of biological processes. This is particularly true for related disciplines that traditionally lack accessible equipment and specialized expertise to enable such studies. We envisage rapid uptake of the R-PNAI-T as a companion kit to evaluate the feasibility of SPR [35] and validate ChIP-Seq [80] studies.

## Supplementary Material

gkag142_Supplemental_Files

## Data Availability

The data underlying this article will be shared on reasonable request to the corresponding author.
