# Supplementary material for: A rapid test for protein–DNA interactions
Source: Nucleic Acids Res. 2026 Feb 18;54(4):gkag142. doi: 10.1093/nar/gkag142 (PMC12914326; doi:10.1093/nar/gkag142)
Supplement: gkag142_Supplemental_Files [file gkag142_supplemental_files.zip › Toft 2025 ESI main with Biorender credit.pdf]

A RAPID TEST FOR PROTEIN-DNA INTERACTIONS

Casey J. Toft, Holly M. Radford, Alanna E. Sorenson, Patrick M. Schaeffer*

Biomedical Sciences and Molecular Biology, College of Medicine and Dentistry, James Cook University, Douglas, QLD, 4811, Australia

* To whom correspondence should be addressed. Tel: +61 (0) 7 4781 4448

Email: patrick.schaeffer@jcu.edu.au

ORCID: 0000-0002-0717-5984

SUPPLEMENTARY INFORMATION

Table S1. Resources

Reagent	Description	Notes
**Antibodies**		
$_{fluo}$Anti-GFP IgG	Polyclonal goat IgG conjugated with FITC (3.5 moles FITC per mole of goat IgG).	Ab6662, stock = 1 mg/mL
$_{biotin}$Anti-GFP IgG	Polyclonal goat IgG conjugated with biotin (10-20 Biotin molecules per goat IgG molecule).	Ab6658, stock = 1 mg/mL
**Oligonucleotides**		
$_{biotin}$*Ter1*	5′[Bio]AAAAAAAATATTAGTTACAACATACTGTTT3′<br>3′ TATAATCAATGTTGTATGACAAA5′	Annealed at 10 μM*
$_{biotin}$*Ter1*-lock	5′[Bio]AAAAAAAATATTAGTTACAACATACTGTTT3′<br>3′ TATAATCAATGTTGTAT 5′	Annealed at 10 μM*
$_{fluo}$*Ter1*-lock	5'[FAM]AAAAAAAATATTAGTTACAACATACTGTTT3′<br>3′ TATAATCAATGTTGTAT 5′	Annealed at 10 μM*
Ter1	5' CCCCGCCCCCATATTAGTTACAACATACTGTTT3′<br>3′ TATAATCAATGTTGTATGACAAA5′	Annealed at 10 or 50 μM*
Ter1-lock	5' CCCCGCCCCCATATTAGTTACAACATACTGTTT3′<br>3′ TATAATCAATGTTGTAT 5′	Annealed at 10 or 50 μM*
$_{biotin}$*bioO*	5′[Bio]TAATCGACTTGTAAACCAAATTGAAAAGATTTAGGTTTACAAGTCTACAC3′<br>3′ ATTAGCTGAACATTTGGTTTAACTTTTCTAAATCCAAATGTTCAGATGTG5′	Annealed at 10 μM#
PCRa primer (F)	5′[Bio]TCTGTGGATAACACCCAA3′<br>Coordinates: 4074260 - 4074277 (+)strand	Sequence ID: BX571965.1
PCRa primer (R)	5′CCGATTCTGTGGATAACTT3′<br>Coordinates: 4074446 - 4074428 (-)strand	Sequence ID: BX571965.1
PCRb primer (F)	5′[Bio]GGCCGCGCGGGAGTTA3′<br>Coordinates: 87142 - 87157 (+)strand	Sequence ID: BX571965.1
PCRb primer (R)	5′ACGCAATCGCCGAAAATAACA3′<br>Coordinates: 87326 - 87306 (-)strand	Sequence ID: BX571965.1

* in PBS which was prepared by dissolving a phosphate buffered saline tablet (Sigma Aldrich, P4177) in 200 mL water.

# in annealing buffer: 10 mM Tris pH 8.0, 150 mM NaCl

Expression and purification of proteins

The $_{Dp}$TusGFP (1), $_{Ec}$TusGFP (1),$_{Bp}$DnaG-GFP (2,3) BirA-GFP (4), SARS-CoV-2 NP-GFP (5) and MERS-CoV NP-GFP (5) were produced in *E. coli* BL21(DE3)RIPL and purified as previously described.

The *dnaA* gene from *B. pseudomallei* (K946243 strain) was codon optimized and synthesized for expression in *E. coli* (Bioneer), and cloned into pIM013 vector (6). Briefly, transformed KRX cells in SOC medium were streaked onto LB agar plates

supplemented with ampicillin 100 µg/mL and 0.2% glucose and incubated overnight at 37ºC. Single colonies were streaked on a master plate and grown for a further 24 hours at 37ºC. The bacterial lawn grown from a single colony was used to inoculate 100 mL of TB with ampicillin (100 µg/mL). The culture was incubated while shaking at 200 rpm overnight at 37ºC, until an OD_{600} of 1-1.5 was reached. Overexpression of $_{Bp}$DnaA-GFP was then induced with the addition 0.1% rhamnose and an additional 100 µg/mL of ampicillin. Expression cultures were incubated shaking for 24 hours at 16ºC before cells were harvested via centrifugation. The $_{Bp}$DnaA-GFP was then purified similar to TusGFP with a modified protein pellet resuspension buffer (PBS, 5% glycerol, 2 mM ß-mercaptoethanol, pH 7.2). Protein concentrations were determined by Bradford Assay and by GFP-fluorescence and the purity assessed by SDS-PAGE. Prior to their use in each assay, the fluorescence of all GFP-tagged protein suspensions were checked at 1 µM for quality control (BMG LABTECH microplate reader, ex485, em520, 1500 gain).

Genomics: Identification of DnaA boxes

The approximate location of the origin of replication for *B. pseudomallei* chromosome 1 (K94623 strain) was identified by GC skew analysis with a window and step size of 10-kbp. Two *OriC* candidates were then identified using sequence matches of the *E. coli* 9mer DnaA box (TTATCCACA) around the point of inflection of the GC skew and upstream of the *dnaA* gene. The search included DnaA box sequences with up to two mismatches as well as regions of high AT content (>60-80%). Polymerase chain reaction (PCR) was performed to amplify both identified *OriC* candidates (see Resources for primer details).

SUPPLEMENTARY FIGURES

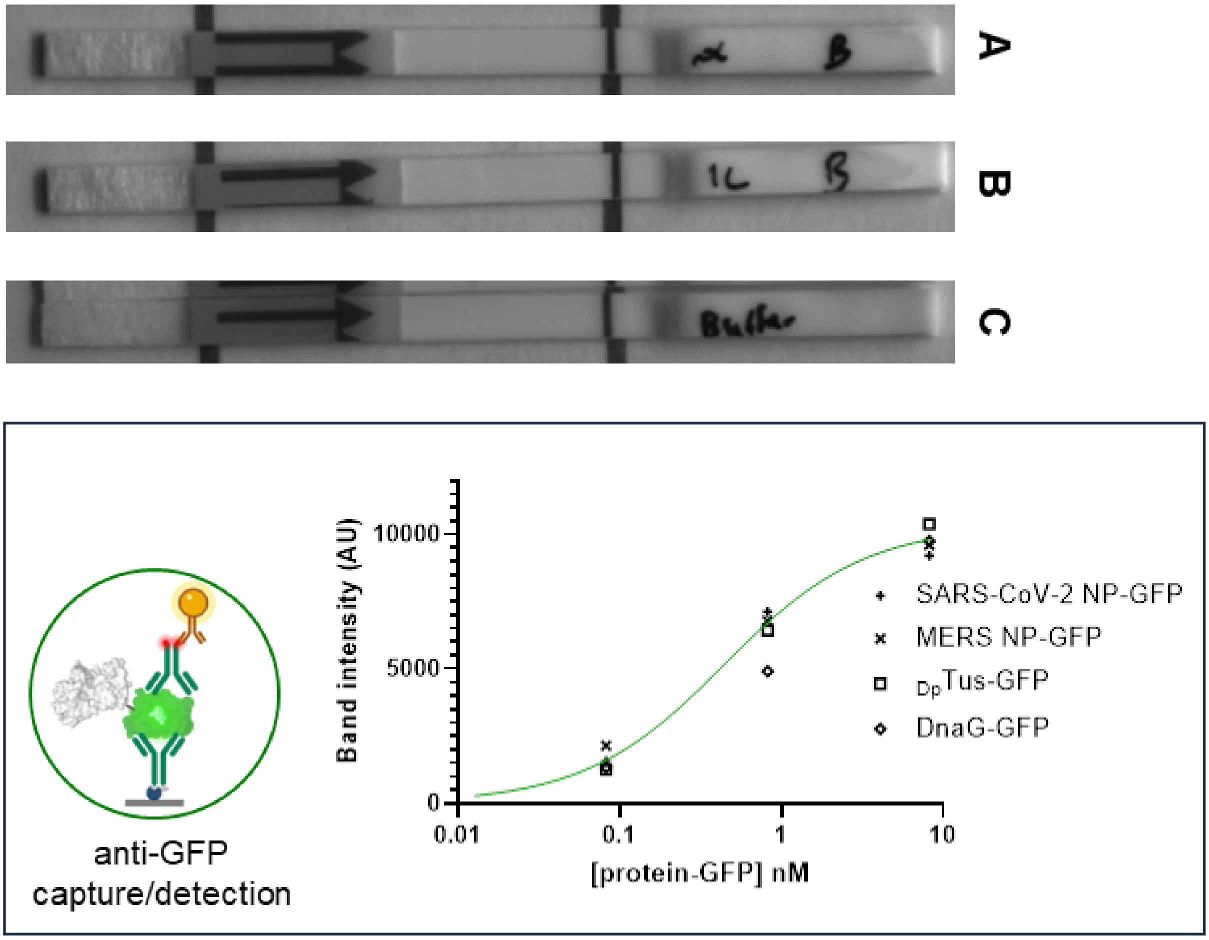

Figure S1. Top panel: R-PNAI-T negative controls: anti-GFP capture/detection format ($_{\text{biotin}}$anti-GFP/$_{\text{fluo}}$anti-GFP) without TusGFP (A), *Ter1*-lock capture format ($_{\text{biotin}}$*Ter1*-lock/$_{\text{fluo}}$anti-GFP) without TusGFP (B) and the *Ter1*-lock capture format without $_{\text{biotin}}$*Ter1*-lock (C). **Bottom Panel:** Detection of various GFP-tagged proteins in similar buffer conditions using the anti-GFP sandwich format ($_{\text{biotin}}$anti-GFP/$_{\text{fluo}}$anti-GFP). Partially created in BioRender. Toft, C. (2026) https://BioRender.com/w35o738.

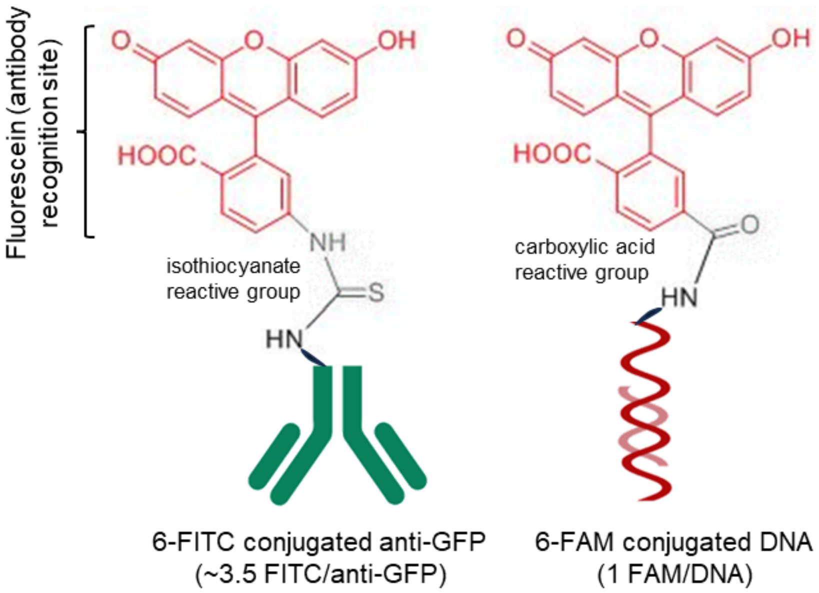

Figure S2. Difference between FITC and FAM.

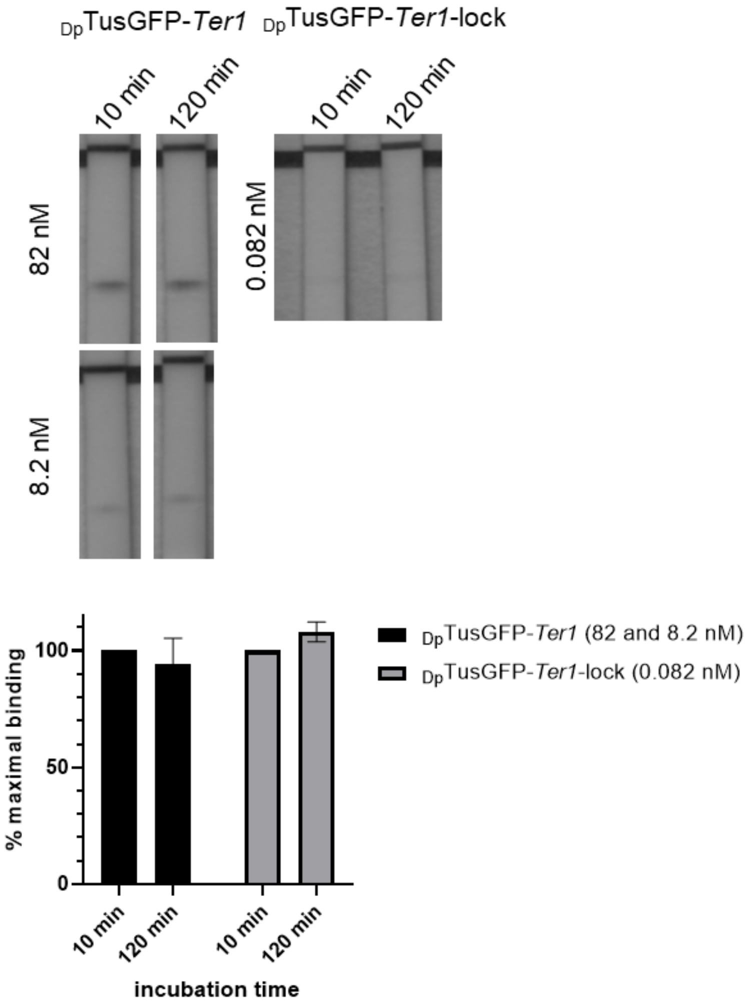

Figure S3. Effect of equilibration time with $_{Dp}$TusGFP-*Ter1* and *Ter1*-lock. Test line band intensities obtained for each condition after a 10 min and 120 min equilibration prior to the addition of the Hybridetect dipstick. DNA (5 pmol) is the same for each experiment and the final protein concentration is varied as indicated.

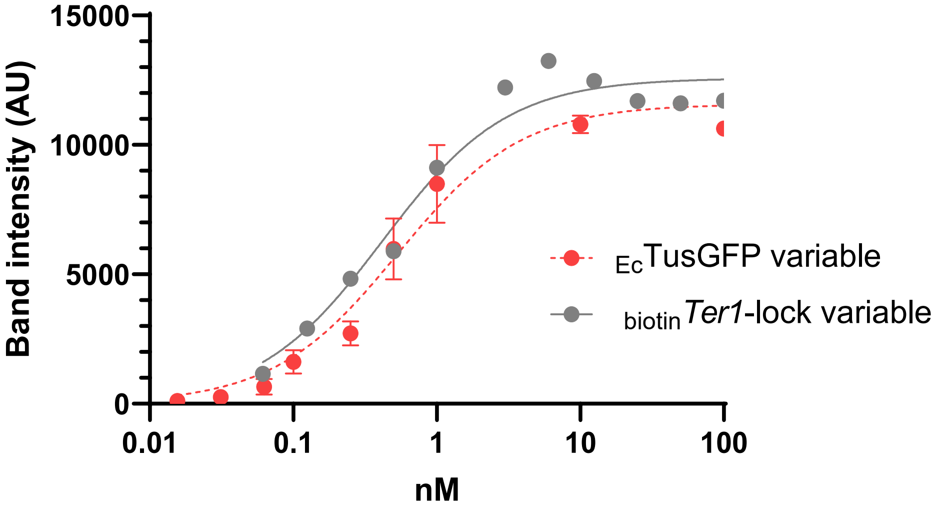

Figure S4. Binding of $_{Ec}$TusGFP to $_{biotin}$*Ter1*-lock with varying concentrations of protein and a constant 91 nM DNA concentration (red) compared to varying concentrations of DNA and a constant 82 nM protein concentration (grey).

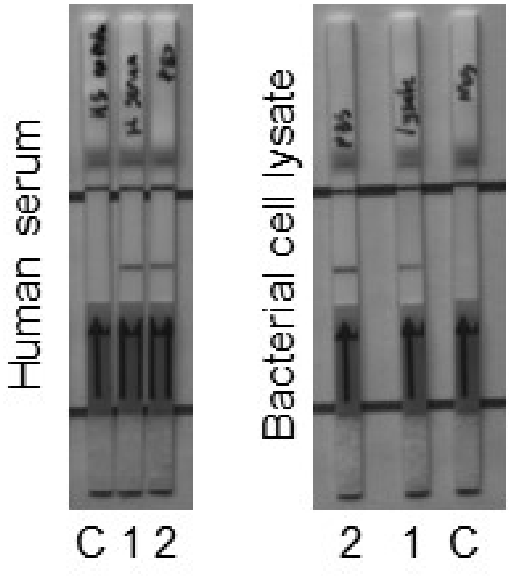

Figure S5. Effect of bacterial lysate and human serum on R-PNAI-T sensitivity. Representative spike-recovery experiments with 50 fmol $_{\text{Ec}}$TusGFP and 5 pmol $_{\text{biotin}}$*Ter1*-lock in neat human serum or bacterial cell lysate (15 ml/g wet bacteria) compared to PBS. Band intensities were quantified using ImageJ and results are shown in Figure 2D. **C:** Negative control (no $_{\text{Ec}}$TusGFP) in human serum or Bacterial cell lysate. **1:** Experiment (50 fmol $_{\text{Ec}}$TusGFP and 5 pmol $_{\text{biotin}}$*Ter1*-lock) in human serum or bacterial cell lysate. **2:** Reference experiment (50 fmol $_{\text{Ec}}$TusGFP and 5 pmol $_{\text{biotin}}$*Ter1*-lock) in PBS.

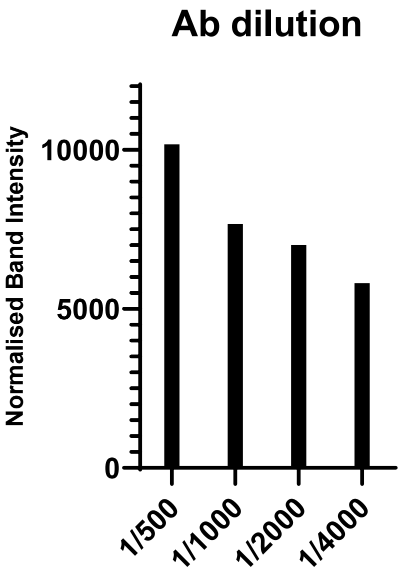

Figure S6. Test line band intensities for 5 nM BirA-GFP with varying concentrations of anti-GFP antibody pair. The effect of dilution was evaluated by combining 20 µL of 10 nM BirA-GFP (diluted in PBS) with 20 µL of a suspension consisting of $_{Fluo}$anti-GFP and $_{biotin}$anti-GFP serially diluted in PBS (1:250 to 1:2000). After 10 min, dipsticks were inserted and data recorded and processed as described in the step-by-step protocol. Final antibody dilution values are indicated.

REFERENCES

1. Toft, C.J., Sorenson, A.E. and Schaeffer, P.M. (2022) A soft Tus-Ter interaction is hiding a fail-safe lock in the replication fork trap of Dickeya paradisiaca. *Microbiol Res*, **263**, 127147.
2. Moreau, M.J.J., Morin, I., Askin, S.P., Cooper, A., Moreland, N.J., Vasudevan, S.G. and Schaeffer, P.M. (2012) Rapid determination of protein stability and ligand binding by differential scanning fluorimetry of GFP-tagged proteins. *RSC Advances*, **2**, 11892-11900.
3. Askin, S., Bond, T.E.H., Sorenson, A.E., Moreau, M.J.J., Antony, H., Davis, R.A. and Schaeffer, P.M. (2018) Selective protein unfolding: a universal mechanism of action for the development of irreversible inhibitors. *Chem Commun*, **54**, 1738-1741.
4. Askin, S.P., Bond, T.E.H. and Schaeffer, P.M. (2016) Green fluorescent protein-based assays for high-throughput functional characterization and ligand-binding studies of biotin protein ligase. *Analytical Methods*, **8**, 418-424.
5. Toft, C.J., Stocks, B.B. and Schaeffer, P.M. (2024) Comparison of the analytical sensitivity of COVID-19 rapid antigen tests in Australia and Canada. *Talanta*, **275**, 126147.
6. Moreau, M.J., Morin, I. and Schaeffer, P.M. (2010) Quantitative determination of protein stability and ligand binding using a green fluorescent protein reporter system. *Mol Biosyst*, **6**, 1285-1292.
