# Supplementary material for: A rapid test for protein–DNA interactions
Source: Nucleic Acids Res. 2026 Feb 18;54(4):gkag142. doi: 10.1093/nar/gkag142 (PMC12914326; doi:10.1093/nar/gkag142)
Supplement: gkag142_Supplemental_Files [file gkag142_supplemental_files.zip › Toft 2025 ESI R-PNAI-T step-by-step guide revision 3.pdf]

A RAPID TEST FOR PROTEIN-DNA INTERACTIONS

Casey J. Toft, Holly M. Radford, Alanna E. Sorenson, Patrick M. Schaeffer*

Biomedical Sciences and Molecular Biology, College of Medicine and Dentistry, James Cook University, Douglas, QLD, 4811, Australia

* To whom correspondence should be addressed. Tel: +61 (0) 7 4781 4448

Email: patrick.schaeffer@jcu.edu.au

ORCID: 0000-0002-0717-5984

GENERAL STEP-BY-STEP PROTOCOL FOR THE R-PNAI-T

This basic procedure can be followed in specialized laboratories as well as educational and low-resource settings. Specific protein-DNA interactions may have unique experimental requirements (e.g. buffer conditions and equilibration times).

Materials:

- 96-well plate with flat bottom
- Hybridetect dipstick (Milenia Biotec).
  - *Maximum binding capacity for $_{biotin}$DNA: 1-2 pmol.*
- Phosphate Buffer Saline (PBS, pH 7.4).
  - *Dissolve one PBS tablet (Sigma Aldrich, P4177) in 200 mL water. Store at 25 °C up to 30 days.*
- GFP-tagged protein diluted in PBS as required.
  - *0.1-100 nM depending on binding affinity of the protein-DNA complex.*
- DNA with 5' biotin modification for streptavidin capture diluted in PBS.
  - *0.1-100 nM depending on binding affinity of the protein-DNA complex.*
- FITC Anti-GFP antibody (abcam, ab6662, 1 mg/ml).
  - *diluted 1:500 in PBS + 20% (w/v) sucrose.*
- Stopwatch or timer
- Camera or smartphone

Estimated cost per test: ~$5 AUD

R-PNAI-T procedure:

For optimal results, a total test volume of at least 40 µL is required, with 55-60 µL being typical. The temperature needs to be kept constant as it impacts affinity and equilibration time.

1. Mix 45 µL of GFP-tagged protein dilution with 5 µL of $_{biotin}$DNA and 5 µL of $_{fluo}$anti-GFP dilutions in a flat bottom well of a 96-well plate.
    - *Allow the sample to equilibrate: 10 min is recommended for scouting experiments but this can be varied depending on the protein-DNA interaction.*
2. Add the Hybridetect dipstick (Milenia Biotec) to the well for 15 min.
    - *During incubation, capillary flow will carry the reaction mixture along the membrane. A distinct control line should become visible, confirming sufficient sample migration time and reagent integrity. If a protein-DNA interaction has occurred, a test line will appear. Test line intensity is indicative of the amount of protein-DNA complex that has been captured.*
    - *Always ensure a control line appears to be sure of test validity.*

Image capture:

Image capture can be performed with a camera, light box or smartphone. For consistency across all images, an 'imaging reference sheet' can be used consisting of an A4 sheet with a dark grey (HEX #A0A0A0) band of ~0.5 cm width printed horizontally across the centre. The sheet can be laminated to create a wipe-clean, reusable surface. The printed line serves as a positional guide for consistent dipstick orientation during imaging as well as a test line standardization during analysis.

1. The 'imaging reference sheet'' is placed on a flat surface and the Hybridetect dipstick positioned with the control line next to the printed grey band (see Figure S7A).
    - *Fixed dipstick placement reduces parallax and makes cropping reproducible and serves as a control for band intensity standardization.*

- *Use a simple light box if available or work in a room with diffuse, shadow free illumination to minimize variation in band intensities due to uneven lighting to simplify image standardization during analysis. Avoid direct overhead spotlights, strong side light and sunlight.*

2. Take photograph with fixed camera and flash turned off.
   - *Lock exposure and focus. Use a fixed camera with the same sample distance ensuring complete field of view so the whole strip fills the frame but is not clipped. Use a small tripod or a book stack to keep distance constant.*
   - *Consistent camera distance during capture is essential for accurate band intensity comparisons between images. Take images in RAW or highest quality TIFF and keep the same image format for all experiments.*

Image analysis:

ImageJ/Fiji program can be used for standardization and band intensity analysis (free to download and use).

1. Convert images to 8-bit grayscale
   - *Open each image and select Image > Type > 8-bit.*
   - *This step simplifies pixel intensity measurements and ensures consistent data type across all images.*
2. Define regions of interest (ROIs)
   - *Use the rectangular selection tool to define a ROI that includes both the test and control lines for each dipstick (see Figure S7A).*
   - *Ensure to define a ROI over the dark grey reference band on the Imaging reference sheet for each image (see Figure S7A). This is used to normalize intensity data between images later.*
   - *All ROIs must have identical width and height across images to ensure valid comparison.*
3. Generate and measure test line intensity profiles
   - *With each ROI selected, use Analyze > Gels > Plot Lanes to obtain intensity profiles (see Figure S7B-C).*

- *Use the Wand tool to delineate the area under each peak corresponding to the control line and test line (see Figure S7D).*
- *Similarly obtain intensity values for the grey reference band from the Imaging reference sheet.*

4. Data normalization
    - *Correct for minor variations in illumination, exposure or saturation using a multiplicative scaling factor to each image so that the grey reference band intensity matches a single chosen target value (we use the median value). This normalization preserves the proportionality and raw intensity scale of the measurements while ensuring consistency and accurate comparisons across datasets.*

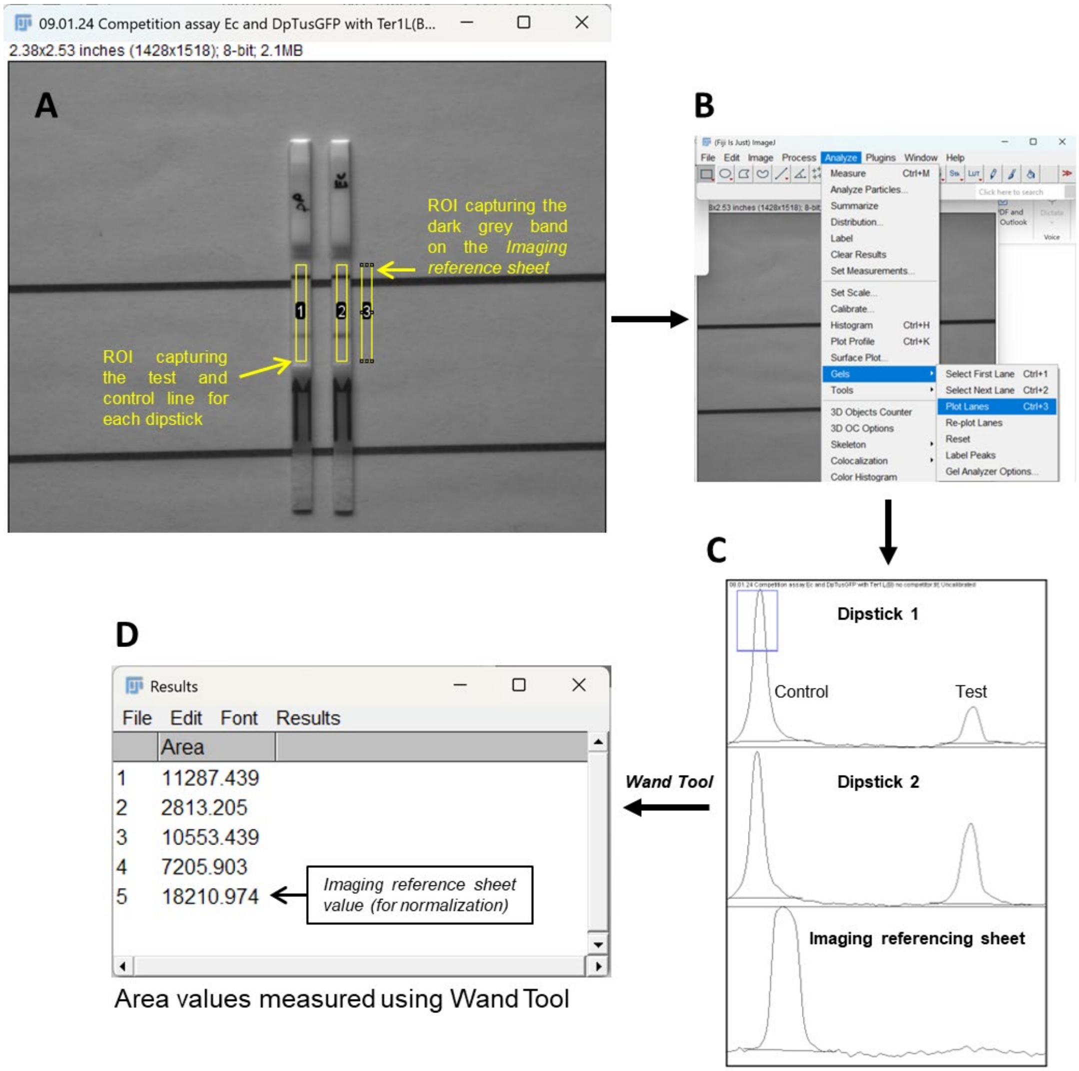

Figure S7. Schematic ImageJ/Fiji workflow for analysis of R-PNAI-T test line results. (A) Defining ROIs for the test/control lines and the grey reference band on the imaging reference sheet. (B) Generating band intensity profiles (C) Example band intensity plots with peaks corresponding to control lines, test lines, and the dark grey line from the imaging reference sheet. (D) Extracted area values corresponding to band intensities; the reference sheet provides a standard intensity for normalization between images.
